# “I Watched my Child Become Half of the Person They Were”: Emergency Remote Learning Experiences Among Primary Caregivers of Elementary School Children During the COVID-19 Pandemic

**DOI:** 10.1177/00220574251349795

**Published:** 2025-06-06

**Authors:** Julia Yates, Cara A. Davidson, Treena Orchard, Shauna M. Burke, Tara Mantler

**Affiliations:** 1 The University of Western Ontario, London, ON, Canada; 2 Children’s Health Research Institute, London, ON, Canada

**Keywords:** achievement, childhood, classroom, learning, teaching

## Abstract

Transitioning to emergency remote learning during the COVID-19 pandemic substantially impacted families; however, the lived experiences of caregivers with young children regarding this transition remain largely unexplored in Canada. The purpose of this paper was to report on how primary caregivers of school-aged children experienced this shift. Semi-structured interviews were conducted with 22 caregivers. Analysis revealed five findings: (1) emotional challenges; (2) home environment-related challenges; (3) gaps in learning; (4) schooling choices; and (5) school supports. Decision makers must ensure that equitable supports that attend to the needs of students and caregivers in relation to emergency remote learning are implemented.

## Introduction

Differing from typical forms of online education (a well-established pedagogical paradigm that comes with unique opportunities and obstacles; Ferri et al., 2020; Gillett-Swan, 2017), emergency remote learning entails a *temporary* and rapid shift in the delivery of education to an alternative delivery model in which all teaching was conducted online due to the global crisis (Hodges et al., 2020). As a result of the COVID-19 pandemic, elementary schools in Ontario fluctuated between in-person and emergency remote learning for the entirety of the 2020–2021 and 2021–2022 school years (totaling nearly 8 months of emergency remote learning; Government of Ontario, 2021).

## Literature Review

Synchronous online teaching can offer benefits over traditional in-person teaching methods, including enhancing elementary students’ motivation (Amasha et al., 2018) and increasing student engagement among those who are uncomfortable participating in traditional settings (e.g., shy or neurodivergent students; Smith et al., 2018). While there are benefits of synchronous online teaching, there are simultaneously numerous challenges including social isolation and fewer opportunities for peer and teacher interaction, which can impact motivational and cognitive outcomes in students (Ferri et al., 2020; Searls, 2012; Zhang & Lin, 2020). In Ontario, synchronous learning was not the reality for many families whose school days predominantly consisted of asynchronous learning during the initial stages of the COVID-19 pandemic. While a firm rationale for asynchronous learning was not provided by the Ontario Ministry of Education, some reasons for this may have included providing increased flexibility to families without access to enough devices or reliable Internet, challenges with teachers having enough time to support each student during synchronous learning, and attempts to minimize distractions associated with synchronous learning (Ontario Public School Boards’ Association [OPSBA], 2021a).

Beyond those impacts that may be experienced while in synchronous learning, asynchronous learning does not provide adequate structure to students, which is especially challenging for elementary learners (Ramadani & Syuraini, 2018), makes providing feedback in a timely manner more challenging, which might result in more students falling behind (Rahman et al., 2022), and does not allow for social connection (Dhawan, 2020). Further, online learning can create technological and financial barriers to education access, particularly for equity-deserving populations who are at greater risk of being left behind in these situations (Tate & Warschauer, 2022). It can also increase the strains experienced by teachers, including the extra time it takes to develop online materials and having to quickly adapt to new online programs for which they may not have received adequate technical training (Recker et al., 2013; Simonson et al., 2009; Tinoca & Oliveira, 2013).

The challenges associated with regulating online education delivery were partially mitigated through the introduction of provincial remote learning requirements for school boards on August 13, 2020 (Government of Ontario, 2023). In this announcement, the Ontario Government outlined the following seven requirements: (1) a schedule of at least 300 minutes of online learning per day, with a combination of synchronous and asynchronous activities; (2) a minimum of 225 synchronous learning minutes for students in Grades 1–8; (3) processes for exemption from synchronous learning; (4) protocols for delivering synchronous learning (e.g., communication processes, differentiated assessment and instruction, supports for students with special education needs, student attendance/safety); (5) access to remote learning devices and the Internet; (6) the provision of a standardized suite of synchronous learning platforms to all boards; and (7) cyber security, privacy, and online safety policies. While school boards were required to report on their fulfillment of these requirements at the end of the school year, to date, there has been no follow-up data publicly released regarding the adherence to or perceived benefits of these requirements.

Despite these seven requirements, concerns were raised surrounding emergency remote learning among elementary students in Ontario and beyond. For example, in a Canadian study of emergency remote teaching during the early stages of the pandemic (i.e., May-July 2020), DeCoito and Estaiteyeh (2022) surveyed Grade 1 to 12 teachers (*n* = 75) and found that 83% of teachers reported challenges regarding the integration of student-centered teaching methods online and 84% reported that they lowered their expectations of student achievement. Further, more than half of teachers (56%) in the study found it difficult to address different students’ needs and academic abilities in an online environment, and most teachers agreed that emergency remote learning had a generally negative impact on student outcomes, including student-student engagement (60%), student-teacher engagement (46%), and student achievement (42%; DeCoito & Estaiteyeh, 2022). Concerns about the negative impact of emergency remote learning on student achievement were founded as school boards have begun reporting the proportion of elementary students meeting grade level reading expectations, with Toronto District School Board (TDSB) reporting a nearly 10-point reduction in the proportion of elementary students meeting grade level reading expectations in 2020–21 when compared to pre-pandemic scores (TDSB, 2021). Although available data beyond the TDSB in Ontario is limited, those collected during the early stages of the COVID-19 pandemic in Quebec (2020-21 school year) showed that 20% of students were failing French and 25% were failing math at that time (Nerestant, 2021). Findings from Alberta echoed those of the TDSB, with students in the second and third grades consistently delayed by six to eight months on their grade level across reading accuracy, fluency, and comprehension measures (Cook, 2020). Based on these reports, it appears that the academic progression of many elementary students in Canada has been impeded as a result of the COVID-19 pandemic.

Beyond academic achievement, the impacts of emergency remote learning on elementary students and their families remain largely unexplored within Canada. Most research conducted to date has been based on teacher perspectives (e.g., DeCoito & Estaiteyeh, 2022) or is based solely on test scores, intending to measure learning gaps resulting from the pandemic (e.g., Cook, 2020; Davies & Aurini, 2021). It is critical to better understand the experiences of elementary-aged children and their caregivers, who had to juggle their own occupational and social transitions alongside the new, often stressful educational needs of their children throughout much of COVID-19 pandemic. To this end, the purpose of this paper was to report on the diverse ways in which primary caregivers of school-aged children (aged 7–10 years) experienced this disruptive shift in their education and home lives during the COVID-19 pandemic. The research question guiding this work was: “How did primary caregivers of school-aged children (aged 7–10 years) experience the shifts in both educational and home spheres during the COVID-19 pandemic?”.

How families experienced shifts in educational and home spheres can be situated within The Family Stress Theory which attempts to explain changes within family systems that occur as a result of stressful events (in this case, the changes caused by the COVID-19 pandemic; Hill, 1966). Based on this theory, each family has both internal and external resources that can buffer or heighten the stressful event, which together influence the family’s perception of the event. These three factors—internal and external resources and family perception—influence the outcome of the stressful event/changes (Hill, 1966). Family Stress Theory purports that conditions such as economic hardship or pressures (e.g., low income, job loss, cutting back on necessities), can cause psychological distress for parents which may impact their parenting practices and, as a result, the wellbeing of children (Gard et al., 2020). Many of these economic hardships or pressures were exacerbated by the precarious position families were in during the COVID-19 pandemic and, as such, it is likely that these may have caused distress within this study’s parent population. By considering the results of this study within the context of this theory, this may help to explain why some families experienced less distress than others, despite facing the same changing educational circumstances within Ontario, Canada.

## Method

This qualitative, cross-sectional study was conducted between February and October 2022. Ethical approval was received from the Non-Medical Research Ethics Board at the host institution (NMREB #119509).

### Participants

Participants were recruited via targeted recruitment ads posted on social media (e.g., Facebook, Twitter, Kijiji) and snowball sampling techniques, wherein participants were invited to share the study details with members of their social networks who also met the eligibility criteria. A total of 22 caregivers participated in this study, an amount that was deemed sufficient based on past research employing interpretive description (Thorne, 2016).

To be eligible for this study, caregivers had to: (1) identify as the primary caregiver (defined as any individual who identified as being primarily responsible for the wellbeing of a child or children) of a child aged 7–10 years; (2) live in Ontario, Canada; (3) have access to an audio-based conferencing software; and (4) understand English. The age range of 7–10 years was selected to capture experiences of both lower- and upper-elementary children (i.e., grades 1–3 and grades 4–6, respectively), while also ensuring that participants’ experiences would not stray beyond the elementary school system, as children older than this may attend middle school which, in Ontario, can be a vastly different experience than elementary school.

All recruitment ads contained instructions asking interested caregivers to email the secure study inbox to confirm their eligibility, to be provided with the online survey link, and to book a convenient interview time slot. Following completion of both the survey and interview, each caregiver was provided with a $40 Amazon e-gift-card in recognition of their time and contributions to the study.

A total of 22 primary caregivers (*M*_age_ = 40.45 years; *SD* = 4.55) participated in this study. Most caregivers identified as female (90.90%), married (86.36%), having a university or college degree (50.00%), and being employed full time (68.18%). Full demographic characteristics can be found in Table 1.

**Table 1.** Demographic Characteristics of Caregivers (*n* = 22) and Children (*n* = 27).

Participant characteristics		
Age (in years) of caregivers, *M* (*SD*)	40.45 (4.55)	
Age (in years) of children, *M* (*SD*)	8.19 (1.14)	
	*n*	%
Gender of caregivers		
Female	20	90.90
Male	2	9.09
Gender of children		
Female	13	48.15
Male	12	44.44
Non-Binary	2	7.41
School grade of children		
Grade 1	2	7.41
Grade 2	9	33.33
Grade 3	8	29.63
Grade 4	5	18.52
Grade 5	1	3.70
Grade 6	1	3.70
N/A	1	3.70
Number of children per family		
1	8	36.36
2	9	40.91
3	3	13.64
4	1	4.55
5	1	4.55
Living situation of children		
Full-time with primary caregiver	22	100
Caregiver marital status		
Married	19	86.36
Common law	1	4.55
Single	1	4.55
N/A	1	4.55
Community type		
Large urban center (100,000+)	15	68.18
Urban center (30,000–99,000)	5	22.73
Rural center (<30,000)	1	4.55
Unsure	1	4.55
Highest education achieved by primary caregiver		
Advanced degree	8	36.36
College or university degree	11	50.00
Some college or university	3	13.64
Employment status prior to COVID-19 pandemic		
Employed full-time	15	68.18
Employed part-time	2	9.09
Casual	1	4.55
Unemployed	3	13.64
Stay-at-home caregiver	1	4.55
Employment status at time of survey completion (i.e., Feb-Oct 2022)		
Employed full-time	13	59.05
Employed part-time	4	18.18
Casual	1	4.55
Unemployed	2	9.09
Seasonal	1	4.55
Freelance	1	4.55
Average annual household income prior to COVID-19 pandemic (CAD)		
< $30,000	3	13.64
$30,000–$59,999	2	9.09
$60,000–$79,999	4	18.18
$80,000–$110,999	3	13.64
$111,000–$150,000	2	9.09
> $150,000	5	22.73
Prefer not to answer	3	13.64
Average annual household income at time of survey completion (i.e., Feb-Oct 2022)		
< $30,000	2	9.09
$30,000–$59,999	2	9.09
$60,000–$79,999	2	9.09
$80,000–$110,999	8	36.36
$111,000–$150,000	1	4.55
> $150,000	3	13.64
Prefer not to answer	4	18.18

*Note.* Data on race/ethnicity is unavailable.

#### Data Collection

##### COVID-19 Context at Time of Study

On March 12, 2020, the Government of Ontario announced that elementary schools would be closed until April 5, 2020, due to the rapid spread of the novel COVID-19 virus (Nielsen, 2020). This re-opening date was extended another two times before the government announced that schools would be physically closed for the remainder of the school year and emergency remote learning would continue (Nielsen, 2020). Elementary students in Ontario experienced nearly 8 months of emergency remote learning across the 2020–2021 and 2021–2022 school years (Government of Ontario, 2021). Although necessary to reduce the spread of the COVID-19 disease, elementary children in Ontario endured more prolonged experiences of emergency remote learning than children living in any other Canadian province or territory (Gallagher-Mackay et al., 2021).

It is important to note that when the interviews began in February 2022, families in Ontario had been given the option to return their child(ren) to in-person learning the previous month. This followed the final emergency remote learning period in Ontario that occurred from January 3^rd^ to January 19^th^, 2022, in response to the rapid spread of the Omicron strain of the COVID-19 virus (Public Health Ontario, 2022). Interviews were conducted until October 2022, following the announcement from Ontario’s Minister of Education, Stephen Lecce, that students would remain in the classroom for the 2022/2023 school year, even if the province experienced a COVID-19 surge in infections in the winter months (Rushowy, 2022). Lecce had pledged a “normal” return to school experience where masks would be optional, physical distancing measures would be non-existent, and isolation requirements would be dropped—all of which did occur in the 2022/2023 school year (CBC News, 2022).

##### Demographics

As part of the online survey component of the larger study, participants’ demographic characteristics were collected, inclusive of age, gender, marital status, geographic location, education, employment status pre-COVID-19 and during COVID-19, household income pre-COVID-19 and during COVID-19, financial strain, number of children, living situations of children, and children’s school board.

##### Interviews

Individual, semi-structured interviews were conducted online via Zoom with each caregiver between February and October 2022. Each interview was conducted by a single member of the research team (i.e., JY; CD; TM) and were approximately 60 minutes in length. All interviews were audio-recorded and transcribed verbatim by a member of the research team, ensuring that each transcript was anonymized prior to analysis.

#### Data Analysis

##### Demographic Information

Measures of central tendency, dispersion, and frequency were calculated for all demographic responses from both caregivers and children.

##### Interviews

Interview data were informally analyzed iteratively as they were collected, such that insights from early interviews were integrated into ongoing data collection and recruitment, as required (Orchard et al., 2014). For example, if the interviewer noticed a certain line of inquiry was soliciting rich data from participants that answered the research question, this would be probed into in future interviews. Additionally, if the interviewers felt certain perspectives were missing from the data (e.g., male-identifying caregivers), recruitment efforts were made to fill this gap. In line with interpretive description methods (per Thorne, 2016), following the completion of all interviews and transcriptions, members of the research team immersed themselves in the data to conduct multiple iterative readings of the interviews to discern a preliminary coding structure. A subset of transcripts was then independently coded using open and axial coding (Williams & Moser, 2019) by a team consisting of two of the five members of the research team, which allowed the researchers to map each interview onto the research questions. Open coding allowed researchers to begin attaching portions of the transcripts to certain codes that were relevant to the research question while axial coding began to develop linkages between codes to create larger categories of findings. Then, each coding dyad, followed by the larger group, met to discuss the applicability and fit of the preliminary coding structure. Refinements to the coding structure were made, as needed. This process was repeated twice, until the research team was confident the codes accurately covered the dataset. Finally, each interview transcript was assigned to two researchers to code for final analysis using Quirkos (2021) online qualitative analysis software.

## Findings

The findings of this research highlight the experiences and perspectives of primary caregivers of school-aged children with regard to emergency remote learning during the COVID-19 pandemic. Caregivers discussed both positive and negative aspects of emergency remote learning experiences, including: (1) emotional challenges; (2) home environment-related challenges; (3) gaps in learning; (4) schooling choices; and (5) school supports.

### Emotional Challenges

Caregivers described the emotional challenges their children experienced related to in-person schooling disruptions during the COVID-19 pandemic and the differences in experiences between older (i.e., 9- and 10-year-olds) and younger (i.e., 7- and 8-year-olds) children.

#### General Emotional Toll

Most caregivers noted how emotionally taxing emergency remote learning was for their children. One caregiver shared the effects of emergency remote learning on their child, saying, “I watched my daughter become half of the person she was. I mean to just listen to her cry about going to do online learning…it was heartbreaking” (P19). According to caregivers, the psychological impacts were experienced by all family members as schooling became a more significant part of the family context, with many caregivers becoming deeply involved in the schooling process. While the process of learning was emotionally challenging, the frequent oscillation between in-person and online schooling that took place with little notice for children and families added an additional layer of difficulties. When periods of emergency remote learning were extended, caregivers noted that this was even more challenging for their children. For example, one caregiver described their son’s reaction to the decision to remain online following winter break:

I just remember him standing in the yard and he just, he had a snowsuit on and he fell to the ground and was just so sad… I feel like online schooling was like someone took my heart and threw it on the ground and stomped all over it. (P14)

The emotional toll of emergency remote learning on caregivers was palpable during interviews. Adding to these challenging experiences was the reality that caregivers had no control over the situation, were forced to be bystanders watching the impacts of these emergency remote learning decisions on their children, and were ultimately the bearers of bad news to their children when it was announced that schools were to remain online.

#### Differences in Children’s Experiences Depending on Age

Emotionally challenging experiences of emergency remote learning were noted among caregivers with children of all ages, but those who had multiple children described the differences they saw in their younger versus older children. Most caregivers expressed that online learning seemed to be easier on the younger children, as many of them did not have/remember prior experiences of in-person learning. As such, they had nothing to compare to, so they were unaware of the loss and/or what they were missing out on. One mother expressed this, saying that her younger child was not impacted by online school as much as her older child, because “…being home with mom was all they knew and doing virtual school was sort of all they knew” (P17). Another participant shared this sentiment, saying that during periods of in-person schooling when health protections were enforced that, “wearing a mask and keeping your distance and not being able to play so close with other children was normal for my youngest” (P19). However, caregivers shared that older children with previous experiences of in-person schooling felt the transition to emergency remote learning as more emotionally taxing. One participant with children in both lower- and upper-elementary grades described this, saying that they “watched [their] children just go through very different paths of this” (P19), with the path for their older child being far more challenging than that of their younger child.

### Home Environment-Related Challenges

Structural challenges, or those challenges with emergency remote learning resulting from the at-home environment, were described by many caregivers and children. These structural challenges stemmed from the differing physical spaces and technology available to families.

#### Challenges with Physical Spaces

According to many caregivers, physical spaces that were carved out by families to facilitate learning in the home environment presented challenges for children and caregivers alike. One caregiver described these challenges, saying, “We didn’t even have desks. So, we had to make-shift spaces for them to learn in and then they could hear each other and see each other…that was really challenging” (P8). These challenges seemed to be amplified when children within the same household were on different school schedules, as one parent (P14) with three children, all on different schedules, explained:

Sometimes they’d be jealous of the other person, like when the other person was on break, it would be hard for the other one to concentrate because that person is getting snacks and doing different things… [my child] would often be like, well [my other child] just gets way more breaks than me and she was seeing it all day because they were in the same house, so she said it’s hard for me to concentrate when he’s singing in the background because he’s on a break.

The distractions for children extended beyond the other people in their household to what they had available to them that they would not normally have access to at school (e.g., personal toys). One parent grappled with ensuring their child attended school at home despite the many ongoing distractions in the familial environment: “At home, he also has access to his toys and to the bedroom, and so he will just come and take a break, he would lie down on the recliner, or he would start playing with his toys” (P6). Along with the distractions came the feeling of caregivers needing to constantly monitor their children to ensure they were staying on task, which was likely due to the length of time children were required to be on screens for; and, because such devices were typically used previously (i.e., pre-pandemic) for entertainment purposes. One caregiver (P8) shared that they modified their physical space to ensure this monitoring could occur, explaining that:

We ended up having to put them on the inside of the “U” so we could see what they were doing, because we noticed she wasn’t concentrating. She was on video games, or watching YouTube videos, or finding other ways to entertain herself.

Other caregivers shared these sentiments, expressing that the constant need to monitor children caused feelings of suffocation for themselves and an overall lack of freedom during the school day for caregivers.

#### Challenges With Technology

Challenges with technology related to navigating online leaning for their kids were commonly expressed by caregivers. Since children in this study were younger and, generally, less experienced with technology than parents or older kids, resolving the technological issues generally fell on the caregivers. Most teachers and school support staff made technology and allied devices available to families, but the assumption from caregivers’ perspectives was that caregivers and children had the knowledge to use and troubleshoot these devices. One caregiver described their struggles with this situation, saying: “The beginning was super rough, super, super rough. We were having trouble logging on. We’d never done like, zoom meetings or anything like half the time the kids’ devices wouldn’t work.” (P5). Other caregivers described feelings of needing to be always “on-call” for their children in case technology issues arose or they needed their assistance (e.g., with handing assignments into online locations). One caregiver shared that, “There were many times when you know, when technically she needed our help. She was like, this is not loading or something’s happening” (P9). Emergency remote learning required access to technology and a reliable Internet service, which posed additional issues for many families who did not have enough devices or Internet bandwidth for all family members who had to be working and attending school at the same time. For families in rural locations, these challenges were exacerbated by the limited options available to them. One caregiver shared that, “We could only access WIFI on one floor, so we all had to kind of navigate that together” (P8).

### Gaps in Learning

Regarding the student learning, or perceived lack thereof, that occurred during emergency remote schooling, the consensus among caregivers was that “there are going to be kids left in the dust” (P22). Whether referring to basic academic skills (e.g., how to read/write) or “life” skills (e.g., how to tidy their things or follow directions), caregivers were largely in agreement that they saw gaps in their child’s learning occurring due to emergency remote learning. These participants were concerned not only with these immediate learning gaps, but also with the potential long-term impacts of this profound disruption in their education for their children. One caregiver (P22) shared:

Now you take two years of disrupted education and that’s really tough to overcome that. And that’s really tough to have to expect the teachers to be able to fix that…you’re going to have kids that are going to be going to school, and they are going to feel stupid, they are going to feel that they’re not good enough, and then they are not going to have the self-confidence to be able to do the things that they need to do because they’re supposed to be in grade five and their only functioning at a grade three level… And I think our kids might not be so bad right now, but you give them four, five, six years of not being able to do the academic work like, what are these kids going to do when they get to high school and they can’t write an essay, because they’re so far behind? (P22)

Other caregivers shared similar feelings stemming from the perceived impact on learning that occurred during their children’s time spent in online schooling. One caregiver shared: “I feel like my child is being denied a public education” (P20). Overall, parents seemed to sympathize with teachers, indicating that they realized that this transition was difficult for everyone and that teachers were often overwhelmed and blindsided with transitions as much as parents were. Nonetheless, having overwhelmed teachers meant for some parents they saw that their children “fall through the cracks a little bit” (P19).

Beyond the academic gaps noted by caregivers, many also indicated that they perceived their children to be missing out on key life skills that were previously taught within classroom settings, such as asking for help or knowing how to tidy up after oneself. One caregiver noted that, “the school teaches them how to organize, how to keep things in place and tidy. She didn’t have that learning… and now everyone’s saying to do things and tidy and she can’t do it because it comes with practice” (P16). For multiple caregivers in this study, the gaps in learning observed among their child (ren) in the public education system during the pandemic was a deciding factor for switching them to a private school or homeschooling them.

### Schooling Choices

The challenges experienced in relation to remote learning meant that many caregivers adapted schooling to better meet their individual family needs. For some caregivers, this meant switching to a form of homeschooling instead of, or while partially participating in, emergency remote learning, while for others this meant choosing the remote schooling option even once in-person options resumed.

#### Adaptations to Emergency Remote Schooling

Many caregivers agreed that emergency remote learning did not work for their children or families. As a result, these participants opted for their personal versions of “homeschooling,” or what some referred to as “unschooling”—which involved some form of adapting or enhancing the schooling offered and was often supplemented by parent-taught lessons. For some caregivers, the reasons behind this choice stemmed from emergency remote learning not seeming challenging enough for their children based on their own perspectives. One caregiver described that, “everything was too easy for my kid, so they hated it, and it was very prescribed, and so we kind of pretended to do that or tried to do that, but we were mostly doing homeschooling” (P19). This caregiver further elaborated that, “for us, home schooling is what’s called unschooling, which is child-led learning” (P19). This allowed their child to explore topics of personal interest while also furthering their education on topics not typically covered in a traditional classroom setting. Other caregivers prescribed to a similar definition of “unschooling,” to allow children to “discover their own interests” (P1). Similarly, another caregiver expressed concerns around what their children were actually learning during emergency remote learning:

We tried and we tried, and you know at the beginning of the week we would get Monday in, and then Tuesday was just a gong show and then by Wednesday I was like screw this you’re not learning anything anyway… What are my kids learning? Like they’re learning how to turn a mic on and off, you know? (P22)

Like others in this study, this family chose to expose their children to various environments where they might learn more “real-world” skills. This caregiver explained, “they were not going to sit there and learn so we went camping… We did crafts, and we went out for hikes. We had to do a lot of learn through play” (P22). Beyond the life skills their children were acquiring through these experiences, caregivers also shared that allowing their children to explore topics of personal interest subsided some of the guilt felt by caregivers that accompanied choosing not to participate fully in emergency remote learning. For other families in the study, the choice to enhance the schooling being offered stemmed from the worry that their children were falling behind in key academic areas. One caregiver explained, “We focused heavily on numeracy and literacy, I think more than the other subjects that the teachers tend to cover, just to try to make sure that their kids weren’t losing too much ground in those key areas” (P11).

#### Remaining Online

For most caregivers who opted to keep their children in remote schooling even after in-person schooling resumed (*n* = 4), it was a choice that stemmed from personal safety reasons, peace of mind, or family illnesses, depending on the family. Many of these caregivers expressed feeling grateful for the option to keep their children home and the ability to make informed decisions about the COVID-19 precautions their families would be taking. For other caregivers, the option of remaining virtual allowed them to “take things one day at a time” (P6). While these sentiments were shared across many caregivers, others had no choice but to opt for virtual schooling for safety reasons. One caregiver shared:

We’re partly choosing to homeschool, and we’re partly forced to homeschool because, and I feel my blood pressure rising because this makes me so angry, you know the Premier and his Minister of Education have done absolutely nothing to support safe school in person. Absolutely nothing. (P20)

Additionally, a caregiver with an immunocompromised child noted, “We’re still the only ones who aren’t doing what everybody else is doing. We’ve had to keep our other kids home from school recently…There’s not a lot of families like ours out there with kids with disabilities” (P1). This caregiver elaborated that sticking the course with online schooling allowed for some semblance of consistency and routine, even amongst the ever-changing circumstances of the COVID-19 pandemic, which, like many others, they found to be helpful for their family.

### School Supports

While many caregivers in this study shared experiences of supportive schools and teachers that helped facilitate emergency remote learning, others shared their disappointment with the supports available to them.

#### School-Wide Supports

Many caregivers indicated that they felt supported when schools acknowledged that emergency remote learning was not going to be perfect, but that they were going to try their best. As one participant said: “Our little one’s school I think did a very smart thing where they acknowledged, they’re not really going to learn as much these next few months” (P7), while another caregiver acknowledged: “the school did what they could with what they had” (P22). Caregivers noted that in some cases, schools went above and beyond to provide additional school-wide supports. One caregiver shared that their child’s school had “emotional support for children. So, they had extra social workers they could talk to, and they had these programs that you can apply for online and like one of them is like CBT therapy for kids” (P4). On the other hand, some caregivers expressed frustration with the lack of support from their child(ren)’s schools: “The school really didn’t do anything to support us, or reach out, or make sure kids were okay” (P8). This includes the perceived paucity of consistent and transparent communication from schools regarding COVID-19, as one participant said “they didn’t want to take a pro or con stand on anything. They were so Petrified of ruffling any feathers… and that was really frustrating that there was no proactivity there because they were so risk averse” (P18). Difficulties with inconsistent or inadequate school communications left some caregivers feeling like schools did not care about their children, with one expressing “that lack of communication, it seemed like a lack of care” (P8).

#### (Un)Supportive Educational Formats

There was a consensus among many caregivers that “teachers did as much as they possibly could” (P22) throughout periods of emergency remote learning. Some teachers were flexible when it came to learning, which caregivers noted helped with online learning to be looked at without “dread and horror” (P7) by their children. When the teachers decreased student workload, caregivers shared that, “it was a way smoother transition because the teachers realized we can’t give the kids this much work. So, it was like a little bit of work, break, a little bit of work, break, which worked out” (P5). Caregivers also shared positive sentiments about teachers who went beyond what was required of them:

The online schoolteachers that we had, they were lovely and wonderful. They went out of their way to do all sorts of stuff, trying to support the kids trying to come up with basically a new way of teaching for that first little bit…I commend them for the, I mean knowing the amount of work that they had to be putting in, they were doing you know, twelve, fourteen hour days every day for months to try to support the kids.” (P13)

While there were many positive accounts of teacher supports by caregivers, some also noted the challenges of the lightened school-work load among children, with some parents feeling like the teachers were not caring, as there were instances where less synchronous time resulted in more independent work. Depending on the age of the child, this meant more work for caregivers who had to monitor and/or assist their children with their learning. One caregiver shared that, from their perspective, online schooling “…[was] a joke. Like they were on three, 30-min sessions a day with the teacher and then they’ve got all this learning on their own and they’re seven” (P14). Another caregiver shared this sentiment, stating that “We were so disappointed. The teacher didn’t do any live lessons at all. There was, there was just nothing” (P8). While some caregivers shared these feelings, it is important to note that most were not blaming the teachers. Those that were synchronous all day—therefore meeting the minimum number of minutes of synchronous activities mandated by the Government of Ontario—shared far more positive experiences than those who were provided mostly asynchronous activities. This was expressed by a caregiver who noted that, “simply trying to port in-person teaching to an online format, I think was a failure” (P7).

#### Technology-Related Supports

Experiences of technology-related supports were vastly different between schools and school boards. Some caregivers shared that they were provided with technology support if needed, including computers or iPads for children to participate in online classes. A few school boards even provided devices with Internet access, to ensure that even families without Internet could participate in emergency remote schooling. Other schools, however, provided suboptimal supports. For one family with three children, they explained that “[They] had to go buy three new Chromebooks because the school gave us one for our three children” (P2). Other families echoed this sentiment, sharing that they ended up having to purchase their own devices because schools were providing them with unusable technology. One caregiver explained:

They gave us basically an old desktop computer, which was really slow… And you know, half the programs that the online school needed couldn’t be installed because it was locked down… [it] became ridiculously untenable. (P13)

## Discussion

The purpose of this paper was report on the diverse ways in which primary caregivers of school-aged children (aged 7–10 years) experienced the disruptive shift in their education and home lives during the COVID-19 pandemic. Findings from this study underscored how emergency remote schooling was both emotionally and structurally challenging for families. Caregivers also highlighted learning difficulties seen among their children and the schooling choices that often accompanied these difficulties. For some, this meant choosing to adapt their own version of “homeschooling” during periods of emergency remote learning while for others, this meant remaining online even after in-person schooling resumed. Supports, or the lack of, stemming from schools, teachers, and technology were also discussed.

Caregivers in the current study shared many challenges their children and families experienced with emergency remote learning, including emotional, structural, and those relating to learning. In an online survey conducted by the OPSBA (2021^b^) on perceptions of online learning in Ontario during the COVID-19 pandemic, similar results were found. This survey (distributed between April-November 2021) collected responses from 8157 individuals, with over 90% identifying as parents (OPSBA, 2021b). Results from this study found that 59% of parents perceived online learning as a largely negative experience for their children, while only 31% perceived the experience positively (OPSBA, 2021b). Based on the Family Stress Theory, it is likely that the families in the current study that perceived the experience positively had both internal and external resources that buffered the stressful changes associated with online learning. Exploring what these resources were that helped families to buffer against changes would be useful in supporting families in future instances of educational change and calls for further collaboration between schools and families moving forward to better support children.

Caregivers with multiple children shared that their older children generally struggled more emotionally than their younger children during emergency remote learning, with some postulating that this might be due to older children having previous experiences of in-person learning to compare to, whereas online learning was the norm for their younger children. These findings contrast those from the OPSBA (2021^b^) survey, which found that more parents supported online learning for Junior (i.e., Grades 4–6) than Primary elementary students (i.e., Grades K to 3). Given that the data in the current study were collected between February and October 2022 and the OPSBA data were collected between April and November 2021, it is possible that the impacts of the ongoing oscillation between in-person and remote schooling compounded for older students later into the pandemic. Based on the current study, ongoing supports with emotional, structural, and learning challenges should be provided *throughout* online learning, versus providing all supports upfront as we typically see, to avoid this compounding of negative impacts on students.

Experiences of online teaching for children and families in the current study were variable. This is relatively unsurprising given the varying emotional challenges, time constraints, and technological resources and proficiencies within classrooms, and what we know from The Family Stress Theory. These variations in experiences likely point to the differences in resource availability between classrooms and schools. Compared to in-person teaching, previous research shows that online teaching routinely proves to be more challenging for teachers as it relates to classroom management (Lathifah et al., 2020), student feedback and assessments (Alsaaty et al., 2016), student interactions (Weizheng, 2019), and technology and planning (Winter et al., 2021). These differences were likely amplified during the pandemic as teachers were contending with the additional stress and trauma of an ongoing pandemic on top of their changing job responsibilities (Dayal, 2023).

Piling onto the additional stress of the pandemic for teachers in Ontario was the combination of the transfer between in-person and emergency remote learning with the lack of notice they were provided when going online. Although the Ontario Government outlined provincial remote learning requirements in August 2020, without proper notice of the transition many teachers had no choice but to use their in-person lesson plans for online teaching. In fact, DeCoito and Estaiteyeh (2022) found that 59% of teachers surveyed in Canada (*n* = 75) felt their online teaching methods were less creative than their in-person teaching methods, while 70% also reported being more content-oriented than teaching-strategy-oriented during periods of emergency remote teaching. Teachers also reported gaps in their technology, pedagogy, content knowledge, and self-efficacy (DeCoito & Estaiteyeh, 2022). Given the lack of teacher training related to online content delivery and lack of time with which elementary teachers had to put their lessons online, many teachers likely had to prioritize delivering content versus integrating student-centered or creative teaching strategies. In future experiences of emergency remote learning, it would be beneficial for school boards, but more importantly the Government of Ontario and the Ministry of Education, to prioritize supports for teachers, including increased professional development opportunities related to online teaching methods and a clearer plan that considers the needs of teachers, students, and families to ensure the quality of elementary education.

Technology supports were also experienced differently by caregivers with children enrolled in different school boards. While some shared that the technology supports provided were above and beyond what they expected, others described them as being woefully inadequate. Later into the pandemic, equity become a more central focus, with schools working to ensure all children had access to computers and lesson plans, while not assuming in-home resources were available to support learning (Public Health Ontario, 2022), in line with the requirements for school boards put forth by the Ontario Government (Government of Ontario, 2023). Despite this, our findings surrounding technology struggles were unsurprising given that research revealed that approximately 13% of households in Canada were without Internet service and large inequities existed between the Internet speeds of rural versus urban households (CRTC; 2020). Some families living in rural locations even described “dead zones” where reliable Internet access was virtually unattainable at any cost (Jabakhanji, 2020). Further, while more Canadian households were purchasing higher Internet speeds in 2019 compared to 2015, these speeds came at a cost that remained unattainable to many families (Frenette et al., 2020). In fact, Frenette and colleagues (2020) found that during the COVID-19 pandemic, those households in the lowest income quartile were significantly less likely to have home Internet and have multiple internet-enabled devices than those in the highest quartile. This likely contributed to the challenges described by caregivers in the current study, especially those of lower incomes who resided in rural locations with unreliable Internet access.

Further pursuant to the technology struggles that many families faced was likely the availability of usable devices. Despite transitioning to emergency remote learning during spring 2020, around 2000 students in the Toronto District School Board were still waiting to receive electronic devices in October 2020 (Samba, 2020). This delay was said to stem from the lack of available devices on the market that followed the abrupt initial transition to emergency remote learning (Samba, 2020). Combined, these results reinforce equity concerns surrounding the impacts of emergency remote learning on disadvantaged populations, especially those of low socioeconomic status and rural geographies. Conscious efforts to ensure responses to future pandemic experiencers are equitable should be at the forefront for decisionmakers to ensure these already disadvantaged populations are not further impacted by avoidable circumstances.

### Limitations and Future Directions for Research

The results of this study should be considered within the context of the study’s limitations. Firstly, the sample was not entirely representative of the broader population in Canada. Specifically, the sample was lacking diverse gender and socioeconomic representation, while also lacking data on ethnicity/race due to a survey error. Given that the pandemic was likely experienced very differently by families, while this study attempted to recruit a representative sample, it is likely the ethical requirements of doing virtual research during the pandemic undermined this goal (e.g., reaching those families without Internet access). It is important to make conscious efforts to recruit diverse populations in future research, especially given in-person data collection is now possible. Secondly, collecting data via qualitative interviews was subject to inherent social desirability biases. To mitigate these biases, the interviewers employed honesty demands (Bates, 1992) at the outset of the interviews, and incorporated various techniques to limit social desirability responses (e.g., asking follow-up questions or asking that participants provide a story or example to illustrate their response; Bergen & Labonté, 2019). Lastly, data was not collected specifically regarding participants’ decisions to send their children back to in-person schooling during the time of our study. As such, participant responses may have been impacted depending on if they were still managing emergency remote schooling at the time of their interview.

### Implications for Practice

Results from this study provide the foundation for various practical recommendations for the education system. Firstly, future instances of emergency remote learning or online learning at the elementary level will require purposeful consideration of equity-deserving groups, specifically those of low socioeconomic status and rural geographies. By, in many cases, failing to provide additional supports to these groups, children in these families faced greater barriers to accessing education than their age-similar counterparts. Educational decision makers must prioritize equity-deserving populations when considering and implementing online learning to ensure all children are provided the same opportunity and access to education. Secondly, caregivers of elementary students must be considered in the context of implementing online learning. While teachers and students are regularly considered when making these decisions, albeit not always in equitable or supportive ways, caregivers are routinely left out of these considerations due to the different spheres in which they operate. However, when the school environment is brought into the home, as is the case with online learning, the additional burden this transition causes often falls onto caregivers. Providing supports with emotional, structural, and learning challenges shared by caregivers in this study must be prioritized in future online learning scenarios. Finally, school boards and the governmental bodies in charge of education must prioritize supports for teachers in developing online teaching materials, including financial and human capital resources, to uphold the quality of elementary education. Without the support of the government, it would be nearly impossible to improve these teaching and learning conditions in future iterations of emergency remote learning.

## Conclusion

Experiences of emergency remote learning impacted all families with children enrolled in school during the height of the COVID-19 pandemic. These experiences proved challenging for most families, with some seeming to struggle more than others—supporting the notions purported by The Family Stress Theory which state that families will experience stress differently depending on their internal and external resources as well as their perceptions of the stressful event. As highlighted by the school supports experienced by some families and the extreme lack of support felt by others, it is evident that equitable supports were not provided to Ontario caregivers and children during emergency remote learning. This study highlights the need for policymakers and decision makers to ensure the voices of caregivers and children are being considered when decisions are being made that directly impact their everyday lives. Further, equitable supports that attend to the needs of teachers (e.g., proper training for online teaching), students (e.g., emotional supports), and caregivers (e.g., workplace flexibility for working/supporting children with learning) must be developed to support teachers and families through future experiences of emergency remote learning.
